# Th17 Cell-Mediated Neuroinflammation Is Involved in Neurodegeneration of Aβ_1-42_-Induced Alzheimer’s Disease Model Rats

**DOI:** 10.1371/journal.pone.0075786

**Published:** 2013-10-04

**Authors:** Jun Zhang, Kai-Fu Ke, Zhan Liu, Yi-Hua Qiu, Yu-Ping Peng

**Affiliations:** 1 Department of Physiology, School of Medicine, Nantong University, Nantong, Jiangsu Province, China; 2 Department of Neurology, Affiliated Hospital, Nantong University, Nantong, Jiangsu Province, China; Universidad de Sevilla, Spain

## Abstract

Neuroinflammation, especially innate immunocyte-mediated neuroinflammation, has been reported to participate in pathogenesis of Alzheimer’s disease (AD). However, the involvement of adaptive immune cells, such as CD4^+^ T lymphocytes, in pathogenesis of AD is not well clarified. Herein, we focus on T helper 17 (Th17) cells, a subpopulation of CD4^+^ T cells with high proinflammation, and show the implication of the cells in neurodegeneration of AD. Amyloid β_1-42_ (Aβ_1-42_) was bilaterally injected into hippocampus of rats to induce AD. On days 7 and 14 following the Aβ_1-42_ administration, escape latency of the rats in Morris water maze was increased, expression of amyloid precursor protein was upregulated, but expression of protein phosphatase 2A was downregulated in the hippocampus, and Nissl stain showed neuronal loss and gliosis in CA1 region. Infusion of FITC-linked albumin in blood circulation and combination with immunostaining of hippocampal sections for RORγ, a specific transcriptional factor of Th17 cells, demonstrated blood-brain barrier (BBB) disruption and Th17 cells’ infiltration into brain parenchyma of AD rats. Expression of Th17 proinflammatory cytokines, interleukin (IL)-17 and IL-22, was increased in the hippocampus, and concentrations of the two cytokines were elevated in both the cerebrospinal fluid and the serum in AD occurrence and development. Compared with intact or saline-treated control rats, AD animals indicated an upregulated expression of Fas and FasL in the hippocampus. Further, the immunofluorescent histochemistry on AD hippocampal sections with NeuN, RORγ, Fas and FasL displayed that Fas was principally expressed by neurons and FasL was predominantly expressed by Th17 cells, and that neuronal apoptosis shown by TUNEL and NeuN double-labeled cells increased. These results suggest that Th17 cells, which were infiltrated into AD brain parenchyma, participate in neuroinflammation and neurodegeneration of AD by release of proinflammatory cytokines and by direct action on neurons via Fas/FasL apoptotic pathway.

## Introduction

Alzheimer’s disease (AD), a neurodegenerative disorder with the most common form of dementia, is pathologically characterized by intracellular neurofibrillary tangles in neurons and extracellular amyloid-β (Aβ) deposition in compact structure between neurons. Aβ is formed from a larger protein named amyloid precursor protein (APP) via breakdown by the enzymes, α-, β- and γ-secretases, and deposited in extracellular plaques known as senile plaques [1]–[3]. The formation and deposition of Aβ is an important cause for neuronal death in vulnerable regions such as the neocortex and hippocampus, which induces behavioral and functional deficits of AD [4]. Injection of Aβ into the hippocampus can induce neurodegenerative changes particularly in the CA1 area and therefore, it imitates both pathological and behavioral characteristics of AD [5].

Pathogenesis of AD is multiple, including genetic, neuropathological and biochemical events. Recently, involvement of neuroinflammation in AD pathogenesis has been largely reported. In AD brain, the damaged neurons and neurites, highly insoluble Aβ peptide deposits, and neurofibrillary tangles provide obvious stimuli for inflammation [6]. While inflammation has been thought to arise secondary to degeneration, recent experiments demonstrate that inflammatory mediators may stimulate APP processing by various means and therefore can establish a vicious cycle to AD progression [7]. Thus, in addition to generation of neurotoxic Aβ peptides and their deposition along with neurofibrillary tangle formation, inflammation may be a third important component which, once initiated in response to neurodegeneration or dysfunction, may actively contribute to disease progression and chronicity [7]. Microglia, the innate immunocytes residing in the brain, have been recognized to play a critical role in neuroinflammatory processes of AD. The abnormal production by glia cells of proinflammatory cytokines, chemokines and the complement system, as well as reactive oxygen and nitrogen species, can disrupt nerve terminal activity causing dysfunction and loss of synapses, which correlates with memory decline and also is the phenomena preceding the neuronal death associated with late stages of AD [8].

In addition to glial cells, adaptive immune cells including T and B lymphocytes are also implicated in inflammatory response in AD brain. In the majority of AD cases, number of T cells in brain parenchyma is increased compared with other cases with non-AD degenerative dementias and controls [9]. In general, due to the presence of blood-brain barrier (BBB), peripheral T lymphocytes are not able to enter brain parenchyma. However, T cells are activated and infiltrate in the brain of AD patients, with the characteristics of disturbed activation and altered numbers of various subsets of T cells in the circulation as well as in the AD brain parenchyma [10]. This means that BBB dysfunctions in AD brain and T cells migrate into brain parenchyma to mediate neuroinflammation. T cells contain several subpopulations different in function. T helper 17 (Th17) cells are a newly defined subset of CD4^+^ T lymphocytes that are distinct from traditional Th1, Th2 and regulatory T (Treg) cells. Th17 cells are highly proinflammatory and induce severe autoimmunity by producing proinflammatory cytokines, including interleukin (IL)-17, IL-21 and IL-22 [11]–[13]. Recent report presents that cytokines (IL-21 and IL-22) generated by Th17 cells and transcriptional factor (RORγ) involved in differentiation of Th17 cells are significantly increased in AD patients [14]. Further evidence regarding role and mechanism of Th17 cells in AD occurrence and development remains to be provided.

A mechanism underlying Th17-mediated neurotoxicity in neurodegenerative diseases such as multiple sclerosis is the binding of proinflammatory cytokines released by Th17 cells to their relevant receptors on neurons, which induces neuronal apoptosis or death [15]. In addition, a direct contact between Th17 cells and neurons in Parkinson's disease (PD) that results in neuronal apoptosis or death is also presented [16]. The direct contact mechanism may be through an interaction of Fas and Fas ligand (FasL). Fas belongs to the superfamily of tumor necrosis factor receptors and is expressed on a wide variety of cell types. FasL, however, is expressed predominantly in activated T cells in both membrane and soluble forms [17]. The occupancy of Fas receptor by its ligand induces apoptosis of cell. We hypothesized that by interaction between the two transmembrane proteins, Fas that exists on neurons and FasL that is on Th17 cells, Th17 cells can directly injure neurons. This hypothesis needs to be clarified.

## Materials and Methods

### Ethics Statement

The animal work done in this study followed the National Institute of Health Guide for the Care and Use of Laboratory Animals and was approved by the Institutional Animal Care and Use Committee of Nantong University.

### Preparation of Aβ_1-42_-Induced AD Rat Model

Four-month old Sprague-Dawley rats (Center of Experimental Animals, Nantong University, China), weighing 220–250 g with males and females, were kept on a 12-hour light/dark cycle, and housed individually with free access to food and water. The rats were randomly divided into four groups: intact, saline, 7-day AD model, and 14-day AD model. Intact rats were bred normally and saline animals took the same operation as model animals except that physiological saline were injected instead of Aβ_1-42_. Before Aβ_1–42_ (Sigma-Aldrich, USA) was used, it was firstly incubated in sterile saline at 37°C for 7 days to allow the change in an assembly state of the peptide with ensuing toxicity [18]. The incubated Aβ_1–42_ solutions generally contain both fibril-like structures and different-sized oligomers [19]. Rats, which had been deeply anesthetized with pentobarbital (55 mg/kg, i.p.) and mounted in a stereotactic frame (David Kopf 902-A, USA), were injected by pressure with the incubated Aβ_1–42_ solution into each side of the hippocampus with the volume of 1 µl containing 4 µg Aβ_1-42_ using the following stereotaxic coordinates: 3.6 mm posterior to the bregma, 2.4 mm left/right to the midline, and 2.8 mm ventral to the bregma [20]. The injection was performed within 5 min and following the injection, the needle remained in the target location for 10 min to avoid the tracer reflux along the needle tract. After surgery, each rat was injected with penicillin (100,000 U) in hindquarter muscle to prevent infection.

### Behavioral Testing

Behavioral testing was performed in Morris water maze by two investigators completely blind to the treatment of the animals. The Morris water maze (Xin Ruan XR-XM101, China), a circular black swim tank (160 cm in diameter and 50 cm in depth) with a small circular escape platform (8 cm in diameter) within it, was filled with warm water (23±1°C) to a depth of 27 cm and the escape platform was submerged 1 cm below the surface of the water. Before obtaining escape latency of rats in the Morris water maze, the rats were given four trials (an alternation of 60 s swim and 30 s rest) per day for two consecutive days to find the hidden platform. Therefore, for the 7-day AD model rats, the 6th day post-Aβ_1–42_ injection and for the 14-day AD model animals, the 13th day after Aβ_1–42_ injection started the trials. Swimming activity of the rats was monitored via a video camera mounted overhead and automatically recorded via a video tracking system. The readout was latency to find the hidden platform, i.e., escape latency.

### Nissl Staining

Rats were perfused with 4% paraformaldehyde (pH 7.4) after anesthetized on day 7 or day 14 post-Aβ_1–42_ administration. The 30 µm-thick coronal sections of the brains were cut in a cryostat (Leica CM 1900-1-1, Germany) after the brains were postfixed in the same fixative for 2–4 h at 4°C. To ensure hippocampal sections were matched between groups, anatomical landmarks provided by the brain atlas [20] were used. The sections were mounted on polylysine-coated slides, dried overnight, rehydrated in distilled water, and then submerged in 1% cresyl violet for about 10 min until the desired depth of staining was achieved. After rinsed in distilled water and dehydrated in graded serried of ethanol, the sections were immersed in xylene, mounted in neutral balsam, and then coverslipped. Nissl-positive cells in the pyramidal layer of medial CA1 were observed for neuronal loss.

### Western Blot Analysis

The hippocampus tissue around needle tract for injection of Aβ_1–42_ or saline was homogenized in an SDS sample buffer that contained a mixture of proteinase inhibitors and the supernatant was collected by centrifuging at 4°C at 12,000 rpm for 15 min. The protein concentration was determined by the DC protein assay from Bio-Rad (Hercules, CA), and 10–20 µg of whole cell lysate was loaded for SDS-PAGE. Electrophoresis was performed and the proteins were transferred to PVDF membranes (Pall, USA) using an electroblotting apparatus (Bio-Rad, USA). The membranes were blocked for 1 h in Tris-buffered saline containing 0.1% Tween-20 and 5% dry milk and were then incubated at 4°C overnight with primary antibodies such as APP (1∶1000, Millipone, USA), protein phosphatase 2A (PP2A, 1∶1000, Cell Signaling Technology, USA), IL-17 (1∶100, Santa Cruz Biotechnology, USA), IL-22 (1∶100, Santa Cruz Biotechnology, USA), Fas (1∶200, Santa Cruz Biotechnology, A-20, SC-1023, USA), and FasL (1∶200, Santa Cruz Biotechnology, N-20, SC-834, USA). They were incubated with the IRDye 800-conjugated affinity purified goat anti-rabbit IgG (1∶5000, Rockland Immunochemicals, USA) for 1 h at room temperature and visualization by Odyssey laser scanning system (LI-COR Inc, USA). Blots were re-probed with the monoclonal mouse anti-β-actin antibody (1∶5000, Sigma-Aldrich, USA), followed by reaction with IRDye 800-conjugated affinity purified goat anti-mouse IgG (1∶5000, Rockland Immunochemicals, USA) to confirm equal protein loading. The molecular weight and relative quantity of the protein bands were determined by an image analysis system (Odyssey 3.0 software).

### Real-Time Quantitative Polymerase Chain Reaction (PCR)

Total RNA of the hippocampus tissue around injection needle tract was extracted with Trizol reagent (Invitrogen, USA) according to the manufacturer’s instructions. Potentially contaminating residual genomic DNA was eliminated with RNAse-free DNAse (Promega, USA). After the RNA content was determined by spectrophotometric analysis at 260 nm, 2 µg of total RNA was reversely transcribed in a 20 µl reaction used for cDNA synthesis with murine myelomonocytic lymphoma virus reverse transcriptase (Promega, USA). The single stranded cDNA was then amplified by real-time quantitative PCR for evaluation of relative gene expression levels. Each 20 µl of reaction mixture contained 1 µl of cDNA, 2 µl PCR buffer, 3.0 mM MgCl2, 0.2 mM of each dNTP, 0.2 µM of each pair of oligonucleotide primers, and 1 U Taq DNA polymerase. Reaction procedures were as follows: an initial step at 95°C for 5 min, 40 cycles of 94°C for 15 s, 64°C for 20 s, and 72°C for 20 s. The data was collected using the instrument’s software (Rotor-Gene software, version 6.0) and relative quantification was performed using the comparative threshold (CT) method after determining the CT values for reference (β-actin) and target genes (IL-17, IL-22, Fas and FasL) according to the 2-ΔΔCt method [21], as described by the manufacturer (User Bulletin). Changes in mRNA expression levels were calculated after normalization to β-actin, a house-keeping gene. To verify the specificity of the amplification reaction, melting curve analysis was performed. The primer sequences used in this study were as follows: 5′-TGGACTCTGAGCCGCAATG-3′ (forward) and 5′-GGCGGACAATAGAGGAAACG-3′ (reverse) for IL-17; 5′-AGCGGTGAT GACCAGAACA-3′ (forward) and 5′-CTCAGGGACATAAACAGCAGA-3′ (reverse) for IL-22; 5′-CCCAAGTCCTGAAAGTGTG-3′ (forward) and 5′-CTTCCCGTGAGATTGATACC-3′ (reverse) for Fas; 5′-TGGAATGGGGTTAGGAATGTATC-3′ (forward) and 5′-TTTGGTTTCAGAGGGTGTGC-3′ (reverse) for FasL; and 5′-CGTTGACATCCGTAAAGACC-3′ (forward) and 5′-TAGAGCCACCAATCCACAC-3′ (reverse) for β-actin.

### Measurement of BBB Permeability

Carotid artery of rats was exposed and cannulated with a 24-gauge polyurethane catheter after the rats were anesthetized with sodium pentobarbital (55 mg/kg, i.p.). FITC-labeled albumin (Sigma-Aldrich, USA) was dissolved in buffered saline (10 mg/ml), and the fluorescent dye solution was slowly infused into the carotid artery at a rate of 1 ml/min (10 ml/kg) as previously described [22]. With a three-minute interval after the infusion completion, the rats were killed by decapitation and their brains were fixed for 48 h in 4% paraformaldehyde in 0.05 M phosphate-balanced solution (PBS, pH 7.4). The brains were then cryoprotected overnight in a 30% sucrose in PBS, frozen in isopentane (−50°C) and stored at −80°C. Coronal sections (40 µm in thickness) of the hippocampus were cut in the cryostat and collected in 0.05 M PBS. Leakage of FITC-labeled albumin into brain parenchyma was observed under a fluorescent microscope. These sections additionally immunoreacted with anti-RORγ antibody (Sigma-Aldrich, USA) for determination of infiltration of Th17 cells into brain parenchyma.

### Immunohistochemistry

Free-floating hippocampal sections were collected and blocked with 0.3% Triton X-100, 3% goat serum in 0.01 M PBS (pH 7.3) for 30 min. The slices were incubated with primary antibody (anti-RORγ antibody produced in mouse, Sigma-Aldrich, USA; anti-Fas antibody produced in rabbit, Santa Cruz Biotechnology, A-20, SC-1023, USA; anti-FasL antibody produced in rabbit, Santa Cruz Biotechnology, N-20, SC-834, USA; anti-NeuN antibody produced in mouse, Millipore, USA) diluted in 0.01 M PBS (1∶200), which were incubated overnight at room temperature. The sections were then washed in 0.01 M PBS and incubated with secondary antibody Alexa Fluor 350-conjugated goat anti-rabbit IgG (Jackson, USA) or FITC-conjugated goat anti-mouse IgG antibody (Sigma-Aldrich, USA) diluted in 0.01 M PBS (1∶200) for 4 h at room temperature. After rinsing in 0.01 M PBS, the sections were stuck to glass slides and observed under a fluorescence microscope.

Terminal deoxynucleotidyl transferase-mediated deoxy-UTP-fluorescein nick end labeling (TUNEL) was performed using In Situ Cell Death Detection Kit (Roche Applied Science, Mannheim, Germany) according to the manufacturer’s instructions. After brain sections were stained with NeuN, as mentioned above except that the second antibody was changed to Alexa Fluor 594-conjugated goat anti-mouse IgG (Cell Signaling Technology, USA), the sections were rinsed in PBS and incubated for 60 min at 37°C with 50 µl of TUNEL reaction mixture. After washing with PBS, the slides were analyzed with the fluorescence microscopy.

### Enzyme-Linked Immunosorbent Assay (ELISA)

Blood was taken from right ventricle of rats on the 7th or 14th day after Aβ_1-42_ injection into the hippocampus, and the serum was collected by centrifugation at 3000 rpm for 20 min. Cerebrospinal fluid (CSF) of the rats was withdrawn by foramen magnum puncture. The serum and CSF were frozen and stored at −80°C refrigerator until use. Concentrations of the target cytokines (IL-17 and IL-22) in the serum and CSF were measured by ELISA kits (eBroscience, USA; R&D Systems, USA) according to the manufacturers’ guidelines.

### Statistical Analysis

Data were expressed as means ± standard deviation (M ± SD). Statistical analyses were performed with the Statistics Package for Social Science (SPSS, 12.0). The data were subjected to the one-way analysis of variance (ANOVA), followed by Student-Newman-Keul’s test to compare the data of all groups relative to each other. Differences were considered statistically significant at p<0.05.

## Results

### Aβ_1-42_ Injection in Bilateral Hippocampus Induces AD-Like Changes of Rats

**Figure 1:**
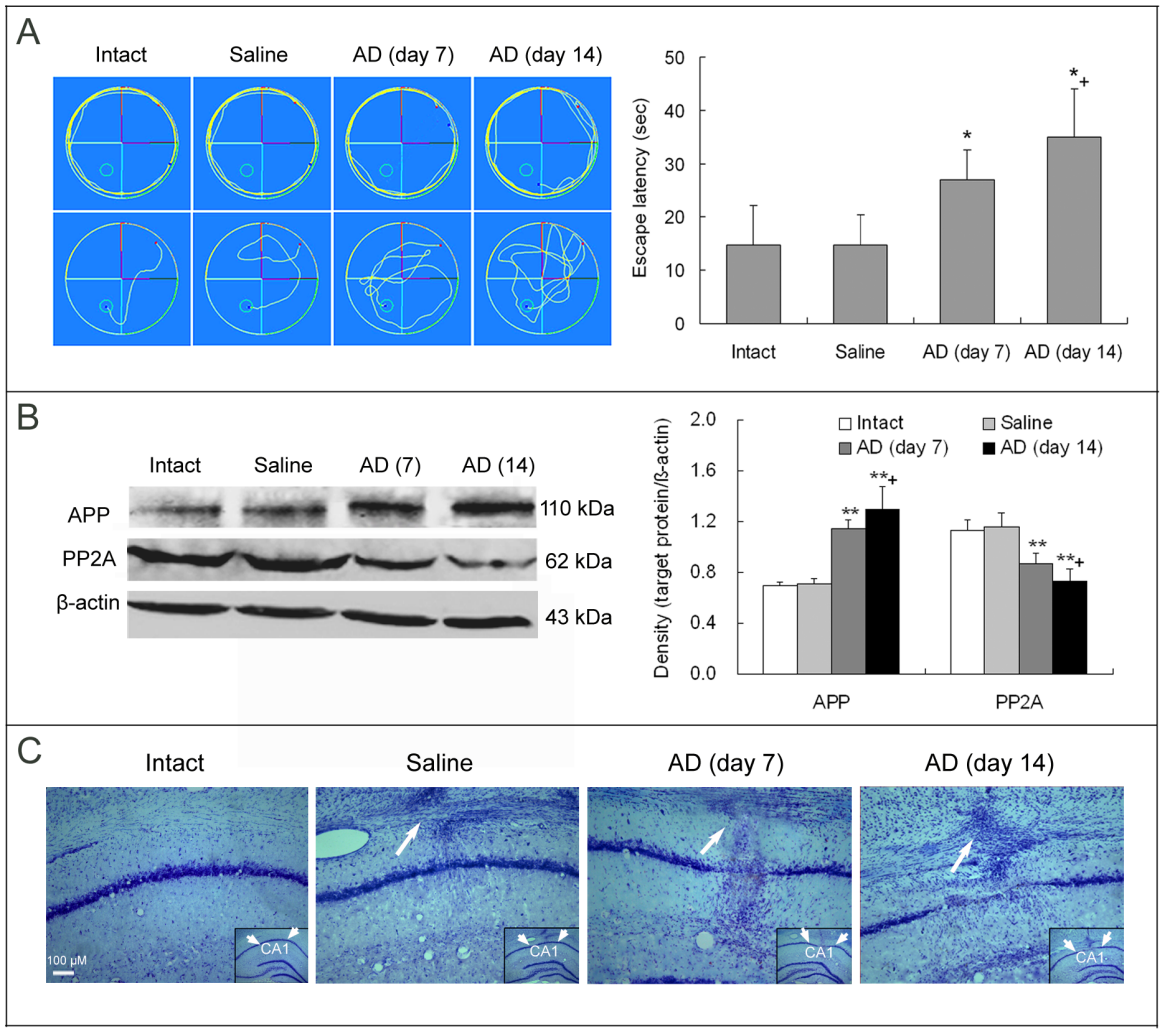
Aβ_1-42_ treatment of bilateral hippocampus of rats induces AD-like changes. Rat hippocampus received 1 µl injection containing 4 µg Aβ_1-42_ on each side. As a control, equal volume of saline was bilaterally injected into the hippocampus. (**A**) Escape latency in water maze for testing spatial learning and memory of rats. Left panel exhibits swimming tracks of rats in Morris water maze. Within the big rounds, the small rounds are escape platforms. The red dots indicate the starting points of the rats, and the blue dots denote their ending points. The upper row displays the first training tracks of the rats. We see that the rats, regardless of treatments, could almost not find the escape platforms within 60 s. The lower row is the final escape tracks of the rats in the water maze after eight trials, which presents that the rats with the Aβ_1-42_ treatment need longer time to reach the hidden platforms. The statistics for seven-repeated experiments is shown in right panel. (**B**) Expression levels of APP, the precursor protein of Aβ, and PP2A, a protein phosphatase that can reduce hyperphosphorylation of tau protein, in the hippocampus. Three or four repetitions were conducted in this experiment. (**C**) Nessl stain of hippocampal CA1 region of rats. We note that neurons lost and glial cells proliferated in the CA1 region where Aβ_1-42_ had been injected 7 day or 14 day earlier. The gliosis also occurred around the injection needle tracks, which are indicated by the long arrows. The insets within the images are general views of the hippocampus and the CA1 regions are denoted. *p<0.05, **p<0.01, vs. intact or saline; +*p*<0.05, vs. AD (day 7). AD (day 7)/AD (day 14): on day 7/on day 14 after Aβ_1-42_ injection in the hippocampus.

Aβ_1-42_ was injected into bilateral hippocampus of rats on day 0. After two day-long training, the rats were tested for spatial learning and memory in Morris water maze. Escape latency in the water maze was markedly increased for the rats with hippocampal injection of Aβ_1-42_ compared with those animals of intact or saline injection (Fig. 1a). The delayed escape latency was longer on day 14 than on day 7 post-Aβ injection (Fig. 1a).

In the hippocampus, APP expression was significantly upregulated but PP2A, an enzyme of protein phosphatase that can reduce hyperphosphorylation of tau protein, was notably downregulated after injection of Aβ_1-42_ (Fig. 1b). These changes were larger on day 14 than on day 7 post-Aβ injection (Fig. 1b).

In histopathological observation of hippocampal sections, we noted that Nissl-stained neurons lost in CA1 region where Aβ_1-42_ had been injected 7 or 14 days earlier, and instead glial cells proliferated in the region and around injection needle track (Fig. 1c). In saline-injected hippocampus, only gliosis around injection needle track but no neuronal loss were seen (Fig. 1c).

These above results showed that Aβ_1-42_ injection in bilateral hippocampus of rats induced AD-like changes and also displayed a progressing process of the AD-like changes from day 7 to day 14 after Aβ_1-42_ injection.

### Th17 Cells Infiltrate into Brain Parenchyma through Disrupted BBB in Aβ_1-42_-Induced AD Model Rats

**Figure 2:**
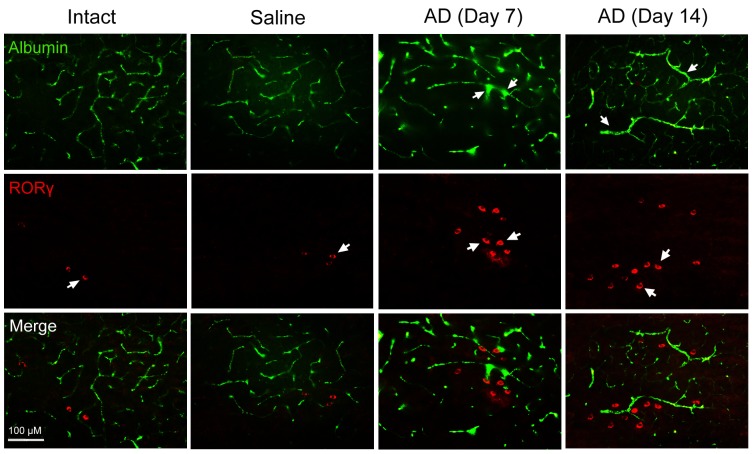
FITC-linked albumin infusion and RORγ immunohistochemistry show BBB disruption and Th17 cell infiltration into brain parenchyma. FITC-labeled albumin was infused into the carotid artery of rats, and then the hippocampus was cut into 40 µm-thick sections for observation of BBB dysfunction. These sections were additionally stained for RORγ, a specific transcriptional factor of Th17 cells. In the upper panel, the arrows denote the effusion of FITC-linked albumin out of blood vessels, suggesting BBB dysfunction. In the middle panel, the arrows point at RORγ-positive cells, which are more in the hippocampus of AD model rats than in that of intact or saline animals, suggesting that Th17 cells increase in AD brain. In the lower panel, we see by the merged images that the RORγ-positive cells are localized around disrupted BBB, suggesting that the Th17 cells infiltrate into brain parenchyma from the disrupted BBB. This experiment was repeated three times and the same phenomena as shown in the photographs were observed. AD (day 7)/AD (day 14): on day 7/on day 14 after Aβ_1-42_ injection in the hippocampus.

By perfusion of FITC-labeled albumin in blood circulation, we observed that the albumin leaked out of blood vessels in the hippocampus where Aβ_1-42_ had been bilaterally injected 7 or 14 day earlier (Fig. 2). It indicated an impairment of BBB in the hippocampus of Aβ_1-42_-induced AD rats. Moreover, number of the immunoreactive cells for RORγ, a specific transcriptional factor of Th17 cells, was increased in the hippocampus with Aβ_1-42_ treatment relative to that in intact or saline-treated hippocampus (Fig. 2). Importantly, these RORγ-positive cells were distributed around the injured blood vessels in the Aβ_1-42_-injected hippocampus (Fig. 2). These results demonstrated that Th17 cells leaked out from the damaged BBB into brain parenchyma of AD.

### Upregulation of IL-17 and IL-22 Levels in the Hippocampus, CSF and Serum of AD Rats

**Figure 3:**
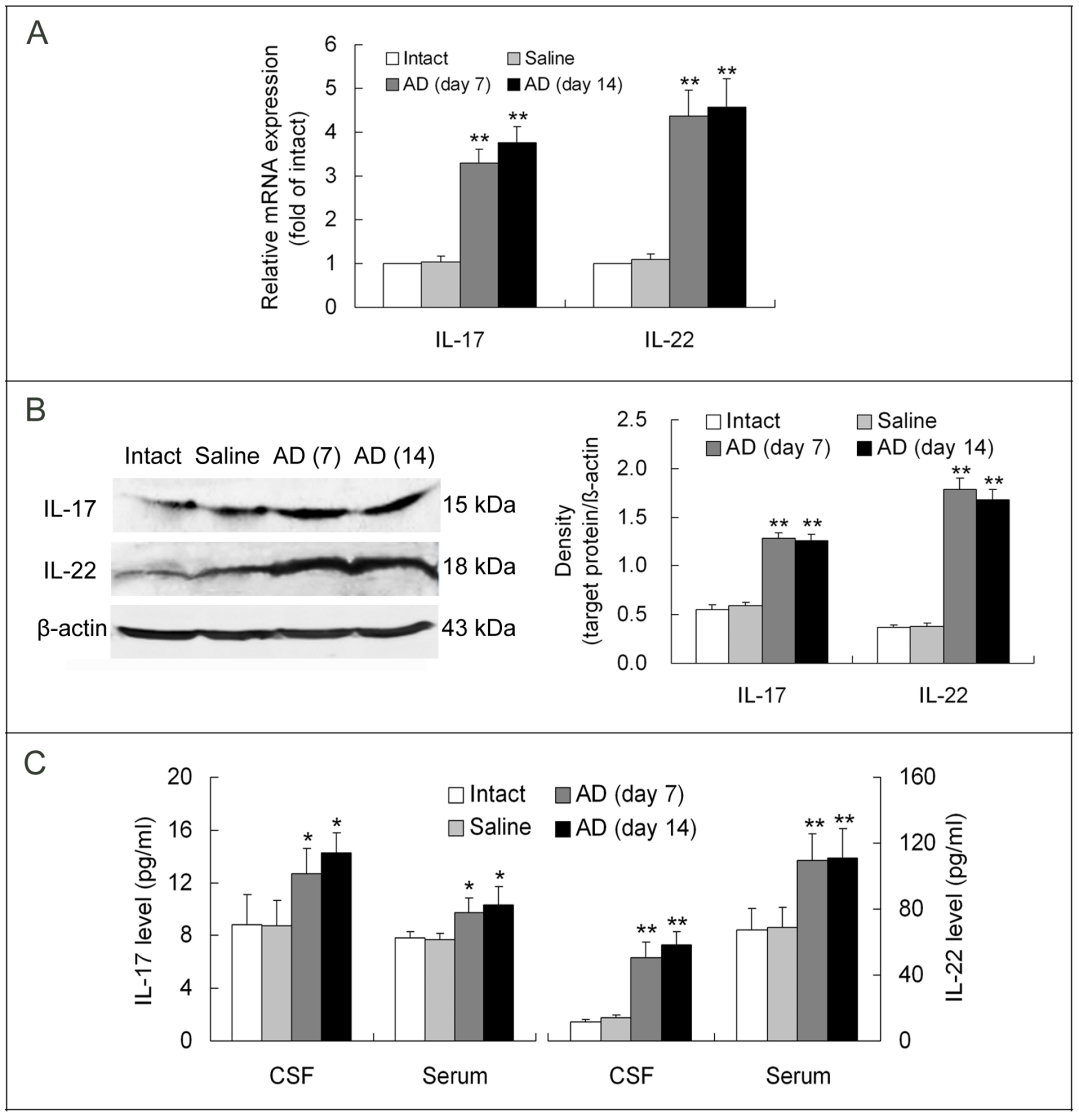
Elevation of IL-17 and IL-22 levels in the hippocampus, CSF and serum of AD rats. The hippocampus tissue around needle tract for injection of Aβ_1-42_ was extracted for measurement of gene and protein expression of Th17 specific cytokines, IL-17 and IL-22. ELISA assay was used to test titers of the two cytokines in the CSF, which was withdrawn by foramen magnum puncture, and in the serum, which was obtained from blood of right ventricle. (**A**) Gene expression of IL-17 and IL-22 in the hippocampus. (**B**) Protein expression of the two cytokines in the hippocampus. (**C**) Concentrations of IL-17 and IL-22 in the CSF and serum. The data are from three- or four-repeated experiments. *p<0.05, **p<0.01, vs. intact or saline. AD (day 7)/AD (day 14): on day 7/on day 14 after Aβ_1-42_ injection in the hippocampus.

To show the involvement of Th17 cell-mediated neuroinflammation in AD neurodegeneration, we determined levels of IL-17 and IL-22, the proinflammatory cytokines produced by Th17 cells, in the hippocampus, CSF and serum of AD rats. Compared with intact or saline-treated rats, AD animals displayed a significant upregulation of IL-17 and IL-22 mRNA and protein expression in the hippocampus and a remarkable elevation of IL-17 and IL-22 titers in the CSF and serum (Fig. 3). Between days 7 and 14 post-Aβ injection, the increased levels of IL-17 or IL-22 in the hippocampus, CSF or serum were similar, and no significant differences were found (Fig. 3).

### Expression and Localization of Fas and FasL in the Hippocampus of AD Rats

**Figure 4:**
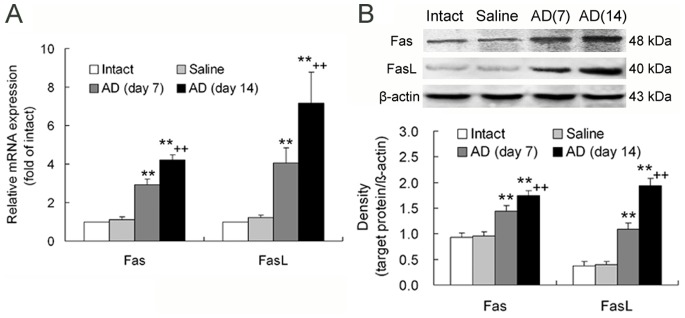
Upregulation of Fas and FasL mRNA and protein expression in the hippocampus of AD rats. The hippocampus tissue around needle tract for injection of Aβ_1-42_ was examined for expression of Fas and FasL, the transmembrane molecules known as death receptor and ligand, respectively. An evidently upregulated gene expression (**A**) and protein expression (**B**) of the two transmembrane proteins is seen. These effects were larger on day 14 than on day 7 post-Aβ_1-42_ injection. The data were obtained from three or four replications of the experiments. **p<0.01, vs. intact or saline; ++*p*<0.01, vs. AD (day 7). AD (day 7)/AD (day 14): on day 7/on day 14 after Aβ_1-42_ injection in the hippocampus.

In the hippocampus of AD rats, Fas and FasL mRNA and protein expression was notably upregulated in comparison with that of intact or saline-injected animals (Fig. 4). The upregulated effect was stronger on day 14 than on day 7 after Aβ_1-42_ administration (Fig. 4), suggesting that more severe apoptosis occurred in AD progression.

**Figure 5:**
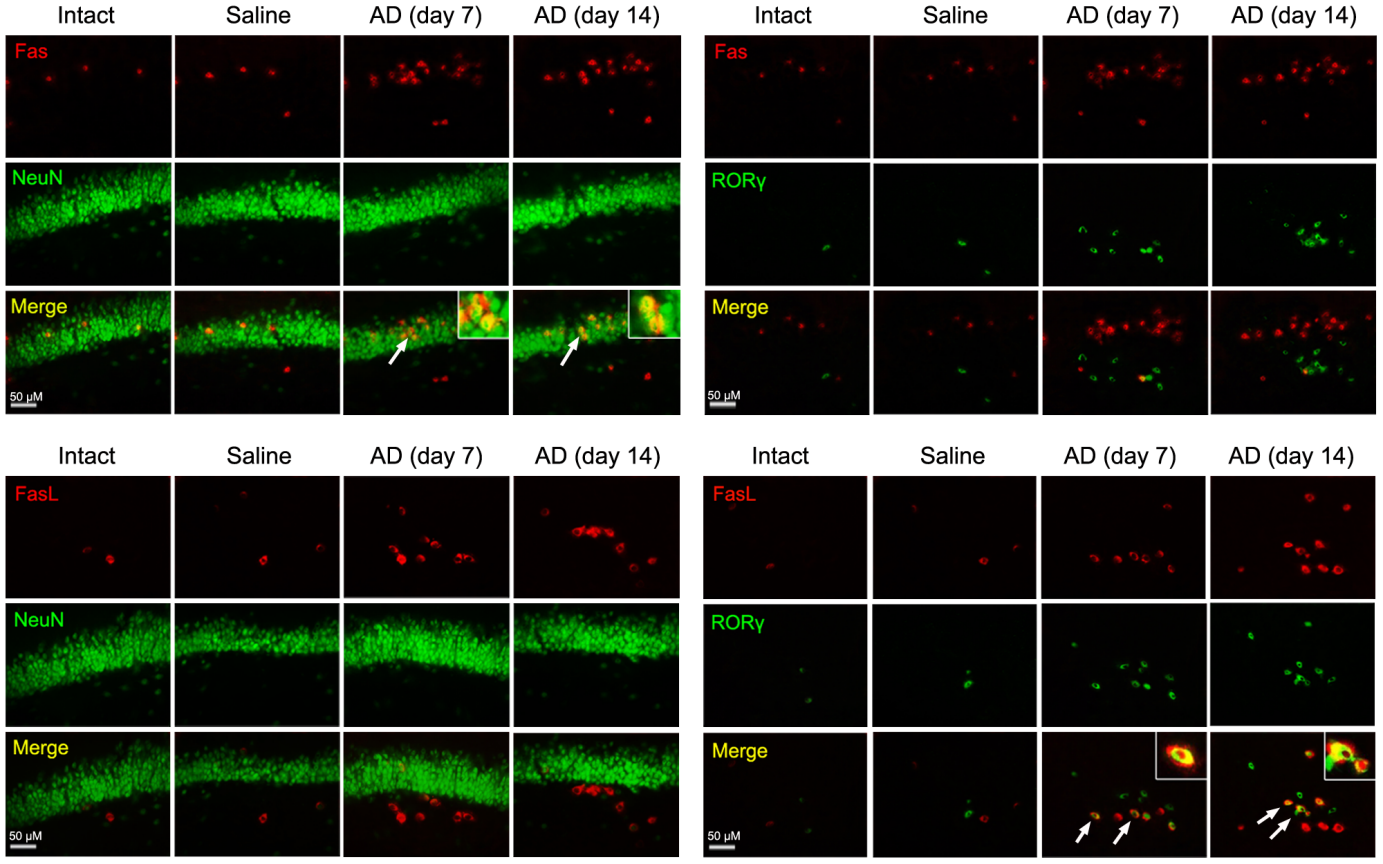
Expression and co-localization of Fas and FasL in neurons and Th17 cells in the hippocampus of AD rats. The hippocampus was coronally cut into 40 µm-thick sections, and these slides were stained with Fas, FasL, NeuN and RORγ by immunofluorescent histochemistry. The immunoreactive cells for Fas, FasL and RORγ are all increased in AD hippocampus, despite the two phases, on days 7 and 14 after Aβ_1-42_ administration. Fas is more co-localized with NeuN but less co-localized with RORγ. On the contrary, FasL is less co-localized with NeuN but more co-localized with RORγ. The arrows point at the double-stained cells, which are magnified in the insets.

To reveal a direct contact injury to neurons by Th17 cells via Fas/FasL pathway, we observed co-localization of Fas/FasL with NeuN/RORγ in the hippocampus of AD animals. As was shown in gene and protein expression of Fas and FasL, the Fas- and FasL-immunoreactive cells in the hippocampus were augmented in both phases of AD (Fig. 5). Similarly, RORγ-positive cells were also increased in the hippocampus where Aβ_1-42_ had been injected before 7 or 14 days. Notably, Fas co-localized mainly with NeuN, but less with RORγ (Fig. 5). In contrast, FasL co-localized principally with RORγ, but less with NeuN, a specific marker for neuronal nucleus (Fig. 5). These findings demonstrated that Fas and FasL were predominantly expressed in neurons and Th17 cells, respectively, in AD brain.

### Neuronal Apoptosis Increases in Hippocampal CA1 Area of AD Rats

**Figure 6:**
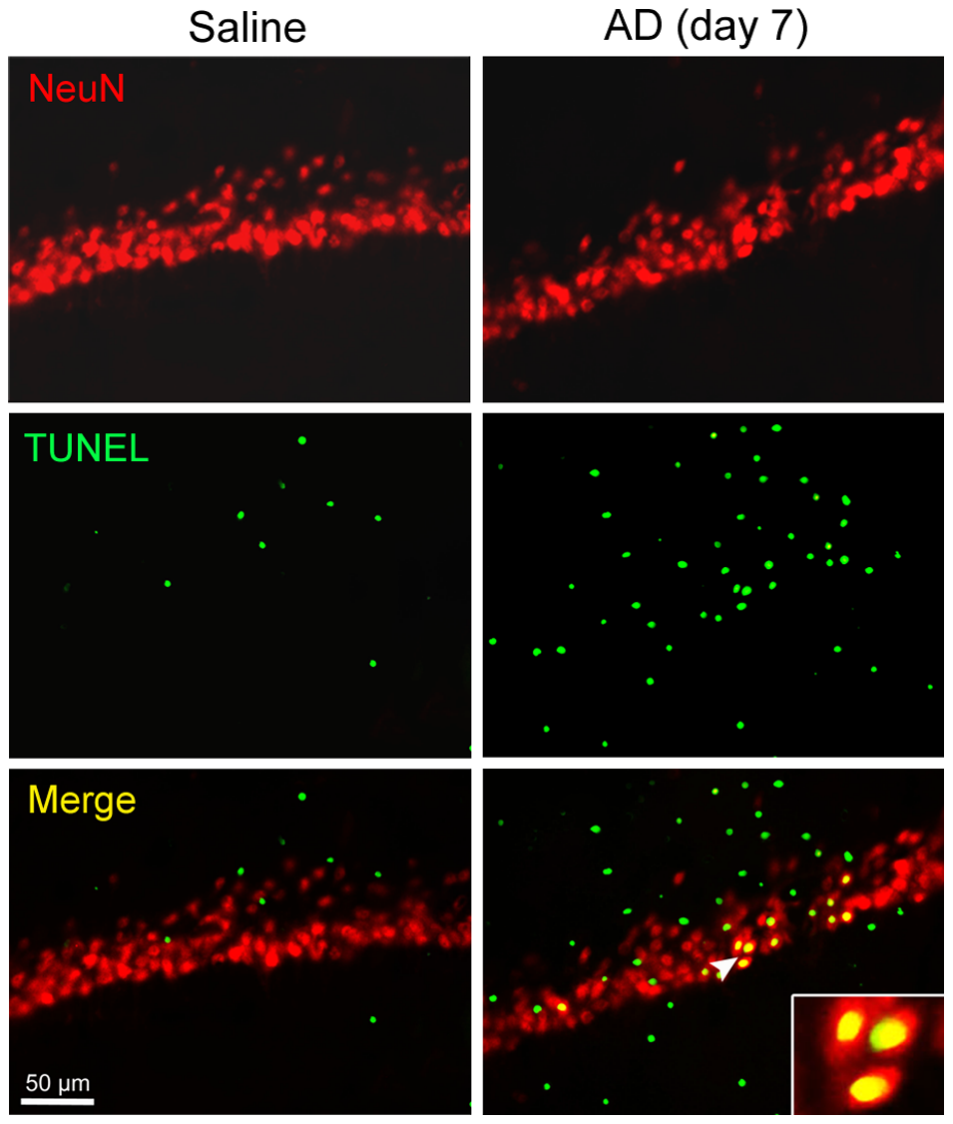
Neuronal apoptosis increases in hippocampal CA1 area of AD rats. TUNEL staining was conducted following NeuN labeling on the brain sections that mainly displayed hippocampal CA1 area, where Aβ_1−42_ had been injected 7 days ago. Note that TUNEL-stained cells increase and that some of them are also labeled by NeuN in the CA1 area of AD hippocampus.

In CA1 area of the hippocampus, where Aβ_1−42_ had been injected 7 days ago, TUNEL-stained cells increased relative to saline-injected CA1 area (Fig. 6). Importantly, there were NeuN and TUNEL double-labeled cells and the NeuN/TUNEL co-localized cells increased in the CA1 area of Aβ_1−42_ injection (Fig. 6). These data showed that Aβ_1−42_ induced neuronal apoptosis.

## Discussion

Hippocampal region is associated with behavioral deficits following injection of Aβ-related peptides [23]. We observed that rats with Aβ_1-42_ injection in bilateral hippocampus had the delayed escape latency in Morris water maze. Moreover, APP, the precursor molecule of Aβ, was significantly upregulated but PP2A, a protein phosphatase that plays a key role in reducing hyperphosphorylation of tau protein [24], was remarkably downregulated in the hippocampus after Aβ_1-42_ administration. More directly, we noted that many neurons lost in CA1 region where Aβ_1-42_ had been injected and instead gliosis occurred in the region. All these effects were more obvious on day 14 than on day 7 following Aβ_1-42_ treatment, suggesting a progressive neurodegeneration in the Aβ_1-42_-induced model rats. Although transgenic animal models for AD have been extensively used, intrahippocampal injection of Aβ_1−42_ in rat brain has been suggested as an animal model which emphasizes the inflammatory reactivity present in human AD brain [25]. Thus, the Aβ_1−42_-induced AD rat model was selected in our present research on neuroinflammation.

In normal brain, BBB prevents peripheral lymphocytes from entering brain parenchyma. In many AD cases, CD3^+^ T cells are more frequent in hippocampal parenchyma than in control cases [9], suggesting a damage to BBB in AD brain, which allows peripheral lymphocytes to enter brain parenchyma. We found that FITC-labeled albumin effused from blood vessels into hippocampal parenchyma of AD rats, supporting a dysfunction of BBB in AD brain. Furthermore, expression of RORγ, a specific transcriptional factor of Th17 cells, was increased in the hippocampus of AD rats, despite the early and late stages of AD. Significantly, the RORγ-immunoreactive cells were localized around the damaged blood vessels. These findings demonstrate that Th17 cells infiltrate into brain parenchyma from the disrupted BBB in AD occurrence and development. In support of our present results, Farkas et al. [26] have indicated that Aβ_25-35_ administration into the right common carotid artery of adult rats induces an enhanced CD3^+^ T-lymphocyte migration towards brain parenchyma due to dysfunctioning of the BBB. Here, the invasion of proinflammatory Th17 cells into the brain provides more evidence for participation in neuroinflammation by the newly defined subset of CD4^+^ T lymphocytes.

Further, expression of IL-17 and IL-22, the proinflammatory cytokines produced by Th17 cells, was significantly upregulated in the hippocampus in the two phases of AD. Similarly, in the CSF and serum of AD rats, IL-17 and IL-22 levels were markedly elevated. The elevation of Th17 cytokines in the brain and in the CSF represents an enhancement of Th17 inflammatory response in the central nervous system (CNS), while the augment of Th17 cytokines in the serum reflects an enhancement of Th17 response in the peripheral tissues. The Th17-mediated inflammatory response enhancement in both the peripheral tissues and the CNS may play a synergic role in promoting AD neuroinflammation and neurodegeneration. Kebir et al. [27] reported that endothelial cells of BBB express IL-17 and IL-22 receptors in autoimmune inflammatory diseases such as multiple sclerosis and that binding to the receptors by IL-17 and IL-22 disrupts BBB tight junctions and facilitates Th17 infiltration into the brain. We presume that this mechanism similarly occurs in AD, by which the increased IL-17 and IL-22 in peripheral blood promotes BBB disruption that allows more Th17 cells to migrate into brain parenchyma to produce more IL-17 and IL-22 in the brain. This synergic effect between the peripheral and central systems aggravates neuroinflammation and exacerbates neurodegeneration of AD.

Fas, the most intensely studied member of death receptor family, is expressed in neurons and distributed on neuronal membrane [28]. FasL, also a transmembrane protein, is expressed in Th17 cells [29]. Fas ligation by FasL is responsible for apoptosis of the Fas-expressing cell by activation of a caspase cascade [30]. In the present study, expression of Fas and FasL mRNAs and proteins in AD hippocampus was significantly upregulated. In support of these results, we found that TUNEL-stained cells increased in AD hippocampus and these cells were labeled by NeuN, a marker for neuronal nucleus. This confirms that apoptosis of neurons occurs in AD progression. Interestingly, although the expression of Fas and FasL both increased in AD hippocampus, the localization of Fas- and FasL-immunoreactive cells was different. Fas had much co-localization with NeuN, but it was less co-localized with RORγ, a transcriptional factor of Th17 cells. On the contrary, FasL had less co-localization with NeuN, but it was greatly co-localized with RORγ. These results demonstrate that neurons upregulate Fas expression and Th17 cells elevate FasL expression in AD brain. Thus, we suggest that a binding of Th17 cell’s FasL to neuronal Fas may be an important mechanism underlying neuronal apoptosis or death directly caused by Th17 cells in AD neurodegeneration. The report presenting that T cells can also directly injure dopamine neurons by signaling through the Fas/FasL system in a chemically induced model of PD [16] supports our present suggestion.

In summary, peripheral Th17 cells infiltrate into brain parenchyma through disrupted BBB in Aβ_1-42_-induced AD model rats. The expression of Th17 proinflammatory cytokines, IL-17 and IL-22, in the hippocampus and the levels of the two cytokines in the CSF and serum are all elevated in AD occurrence and development. These findings provide strong evidence for the involvement of Th17 response in AD neuroinflammation. Simultaneously, expression of Fas and FasL, the transmembrane molecules known as death receptor and ligand, respectively, is significantly upregulated and neuronal apoptosis increases in AD hippocampus. Importantly, the Fas is principally expressed by neurons and the FasL is predominantly expressed by Th17 cells in the hippocampus of AD rats. Accordingly, a direct injury to neurons by Th17 cells via Fas/FasL pathway is suggested.
